# Taxonomy and phylogeny of the lichen genus *Bunodophoron* (*Sphaerophoraceae*, *Lecanorales*) in New Caledonia, with the description of two new species

**DOI:** 10.3897/mycokeys.140.197771

**Published:** 2026-09-11

**Authors:** Nora Helene Borg, Mats Wedin, Einar Timdal, María Prieto, Antoine Simon

**Affiliations:** 1 Department of Biosciences, University of Oslo, P.O. Box 1172 Blindern, NO-0318 Oslo, Norway Department of Biosciences, University of Oslo Oslo Norway; 2 Department of Botany, Swedish Museum of Natural History, PO Box 50007, SE-104 05 Stockholm, Sweden Department of Botany, Swedish Museum of Natural History Stockholm Sweden; 3 Natural History Museum, University of Oslo, P.O. Box 1172 Blindern, NO-0318 Oslo, Norway Natural History Museum, University of Oslo Oslo Norway; 4 Departamento de Biología y Geología, Física y Química Inorgánica, Universidad Rey Juan Carlos (URJC), Tulipán s/n, 28933 Móstoles, Spain Departamento de Biología y Geología, Física y Química Inorgánica, Universidad Rey Juan Carlos (URJC) Móstoles Spain; 5 Instituto de Investigación en Cambio Global (IICG-URJC), Universidad Rey Juan Carlos, Tulipán s/n, 28933 Móstoles, Spain Instituto de Investigación en Cambio Global (IICG-URJC), Universidad Rey Juan Carlos Móstoles Spain

**Keywords:** Australasia, Grande Terre, Melanesia, Nouvelle-Calédonie, Pacific islands, *
Sphaerophorus
*, systematics

## Abstract

This study presents a taxonomic revision of *Bunodophoron* in New Caledonia. Extensive fieldwork was conducted in the region, particularly in the montane rainforests of Grande Terre. An integrative approach was employed, in which specimens underwent morphological assessment, chemical analysis via thin-layer chromatography, and DNA sequencing for four molecular markers: ITS, β-tubulin, MCM7, and RPB1. Phylogenetic analyses were performed within a global framework, incorporating morphologically similar and related taxa from Australasia, the Neotropics, and the Paleotropics, including previously unsequenced taxa analyzed for the first time in this study. Using this approach, four species of *Bunodophoron* lichens were identified in New Caledonia, including two new to science, *B. flagellare* Ant.Simon, N.Borg & Wedin, **sp. nov**. and *B. imbricatum* Ant.Simon, N.Borg & Wedin, **sp. nov**.

## Introduction

Species of *Bunodophoron* A. Massal. are a prominent component of epiphytic lichen communities in cool, wet, and relatively undisturbed forests worldwide (Wedin 1995a). They are particularly noted for their striking appearance, characterized by delicate fruticose thalli that form distinctive tufts. *Bunodophoron* lichens exhibit their greatest documented species diversity in the cool-temperate humid regions of the Southern Hemisphere, particularly in Australasia and southern South America (Kantvilas et al. 1985; Wedin 1995a). In tropical areas, they predominantly occur in high-altitude mountain rainforests (Wedin 1995a).

The *Sphaerophoraceae* (*Lecanorales*, *Ascomycota*), the family to which *Bunodophoron* belongs, is morphologically diverse but has been clearly circumscribed through various phylogenetic studies (Wedin 1993; Wedin and Döring 1999; Svensson and Fryday 2022). Members of the family are unified by a common ascoma development and structure: their densely aggregated medullary hyphae near the ascomatal tissue tend to merge with the surrounding thalline tissue (Döring and Wedin 2000). In addition to *Bunodophoron*, this lineage encompasses the following accepted genera: *Austropeltum* Henssen, Döring & Kantvilas, *Calycidium* Stirt., *Gilbertaria* M. Svensson & Fryday, *Leifidium* Wedin, *Neophyllis* F.Wilson, and *Sphaerophorus* Pers. (Wedin and Döring 1999; Svensson and Fryday 2022; Hyde et al. 2024). With the exception of *Austropeltum*, *Gilbertaria*, and *Neophyllis*, all species have ascomata producing mazaedia (i.e., loose, sooty masses primarily composed of ascospores), which often provide critical diagnostic features for distinguishing closely related species.

*Bunodophoron* differs from *Sphaerophorus* in ascospore shape and formation: *Bunodophoron* produces globose ascospores with an irregular ornamentation of amorphous material that adheres to the ascospore wall after the ascospores are released into the mazaedium. In contrast, *Sphaerophorus* forms broadly ellipsoidal ascospores that develop a thick secondary spore wall while still inside the asci. While sharing similar ascospore features with the genus *Leifidium*, *Bunodophoron* can readily be distinguished from the latter by having relatively dorsiventrally flattened lobes and fruiting bodies that are exposed subapically to ventrally (Wedin 1993). In terms of lichen metabolites, most *Bunodophoron* species, like other members of the *Sphaerophoraceae*, are known to produce orcinol-type depsides (e.g., sphaerophorin), along with a diverse array of secondary compounds, including dibenzofurans and β-orcinol depsidones (Wedin 1995a). However, unlike the morphologically similar genus *Sphaerophorus*, *Bunodophoron* species lack β-orcinol depsides. These chemical profiles often serve as diagnostic tools, complementing morphological examination for species identification.

*Bunodophoron* currently includes 27 accepted species (Wedin 1995a, 1995b; Lumbsch et al. 2011; Soto Medina et al. 2018; Sinha and Jagadeesh Ram 2022). Most of these were originally described under the genus *Sphaerophorus* but were later transferred to *Bunodophoron* following the genus’s reinstatement, resulting in more than 20 new combinations (Wedin 1993). Although the genus is easily recognizable in the field due to its distinct characteristics, its high intraspecific variability can obscure the diversity of these lichens and make species-level identification difficult. Additionally, the diversity of this predominantly temperate genus in the tropics is significantly underestimated due to the relatively unexplored nature of tropical regions in lichenology, especially through molecular techniques. It is therefore likely that *Bunodophoron*, like other charismatic macrolichen genera (e.g., Lücking et al. 2017a, 2017b; Coca et al. 2025), will undergo a substantial increase in recognized species in the tropics as genetic data become available. Recent studies on *Bunodophoron* in the Neotropics using molecular approaches have, as expected, revealed further diversity (Soto Medina et al. 2018).

New Caledonia is recognized as a biodiversity hotspot in the South Pacific, notable for its remarkable diversity of species, including many that are both endemic and relictual (Parkinson et al. 1999; Myers et al. 2000). Despite its modest area of approximately 18,500 square kilometers, the New Caledonian archipelago harbors one of the world’s highest rates of endemism, with an endemism rate of approximately 75% in vascular plants (Morat et al. 2012; Véron et al. 2021). Although the study of lichens in the region dates back more than 150 years (Nylander 1861), recent molecular systematic research on New Caledonian lichens remains limited (Parnmen et al. 2013; Kraichak et al. 2014; Kistenich et al. 2018; Lebreton et al. 2024; Timdal 2025). However, these studies, together with recent comprehensive morphology-based investigations (e.g., Papong et al. 2014), have revealed substantial undocumented diversity, often resulting in the discovery of numerous new species, which underscores the largely unexplored nature of New Caledonia’s lichen flora and its potential for significant new findings. To date, five species of the genus *Bunodophoron* have been reported from New Caledonia (Tibell 1987; Elix and McCarthy 2008), but their identities have not been critically reassessed in the region using both morphological and molecular data. Here, two distinct new *Bunodophoron* species from New Caledonia are described, and some related taxonomic questions remaining in the area are discussed.

## Materials and methods

### Taxon sampling

Most of the recent material treated in the present study was collected on Grande Terre, the largest island in New Caledonia, in 2022 (AS and ET) and 2025 (AS). These specimens were deposited in the lichen herbarium at the University of Oslo (O) and at the Swedish Museum of Natural History (S), with duplicates at the Herbier de Nouvelle-Calédonie (NOU). Additional samples from New Caledonia and other regions, including Argentina, Australia, Chile, Malaysia, and New Zealand, were obtained from various herbaria (B, S, and UPS). These specimens were selected based on their morphological similarity to the New Caledonian samples (Table 1). In particular, outside New Caledonia, the following taxa were examined and included in the phylogenetic treatment, as these were previously reported from the archipelago (Elix and McCarthy 2008; Aptroot and John 2015): *Bunodophoron australe* (Laurer) A. Massal., *Bunodophoron formosanum* (Zahlbr.) Wedin, *Bunodophoron imshaugii* (Ohlsson) Wedin, and *Bunodophoron kinabaluense* (M. Satô) Wedin.

**Table 1.** Voucher information and GenBank numbers for specimens and sequences used in this study. Newly generated sequences are bolded. Previously published sequences were generated by Soto Medina et al. (2018) and Marthinsen et al. (2019). Voucher identifiers correspond either to collector and collection numbers or to herbarium accession numbers (for specimens deposited in O).

**Taxon**	**Voucher**	**Origin**	**ITS**	**β-tubulin**	**RPB1**	**MCM7**
***Bunodophoron agnetae***	**Wheeler & Nelson 820 (S)**	**Chile**				**PZ803587**
***B. agnetae***	**Wedin 9327 (S)**	**New Zealand**				**PZ803588**
*B. australe*	Wedin 8046 (S)	New Zealand		MF954553	MF954576	MF954530
***B. coomerense***	**Wedin 3666 (UPS)**	**Australia**		**PZ803566**	**PZ803600**	**PZ803581**
*B. crespoae*	Soto & Barrera 21I (CUVC)	Colombia		MF954559	MF954582	MF954546
*B. crespoae*	Moncada 45 (UDBC)	Colombia		MF954554	MF954577	MF954535
*B. crespoae*	Moncada 16 (UDBC)	Colombia		MF954555	MF954578	MF954539
*B. crespoae*	Arango 46 (CUVC)	Colombia		MF954557	MF954580	MF954543
*B. crespoae*	Soto & Barrera 20I (S)	Colombia		MF954558	MF954581	MF954540
*B. crespoae*	Moncada 24 (UDBC)	Colombia		MF954556	MF954579	MF954544
*B. diplotypum*	Rivas Plata & Lücking 1195 (F)	Philippines		MF954560	MF954583	MF954532
*B. dodgei*	Wedin 8630 (S)	Argentina		MF954561	MF954584	MF954531
*B. flabellatum*	Hurtado s.n. (CUVC)	Colombia		MF954564	MF954587	MF954537
*B. flabellatum*	Moncada et al. 69 (UDBC)	Colombia		MF954565	MF954588	MF954541
*B. flabellatum*	Benitez & Prieto 4007 (S)	Ecuador		MF954563	MF954586	MF954542
*B. flabellatum*	Benitez, Gonzalez & Prieto 4000 (S)	Ecuador		MF954562	MF954585	MF954548
***B. flagellare***	**Tibell 19906 (UPS)**	**New Caledonia**				**PZ803591**
***B. flagellare***	**L-400469 (O)**	**New Caledonia**	**PZ795345**	**PZ803553**		**PZ803569**
***B. flagellare***	**L-400458 (O)**	**New Caledonia**	**PZ795346**	**PZ803554**	**PZ803592**	**PZ803570**
***B. flagellare***	**L-400465 (O)**	**New Caledonia**	**PZ795347**	**PZ803555**	**PZ803593**	**PZ803571**
***B. formosanum***	**Wedin 3529 (UPS)**	**Australia**				
**B. aff. formosanum**	**L-400514 (O)**	**New Caledonia**	**PZ795348**	**PZ803552**		**PZ803568**
***B. imbricatum***	**Potts s.n. (HO)**	**New Caledonia**		**PZ803564**	**PZ803598**	**PZ803580**
***B. imbricatum***	**L-401096 (O)**	**New Caledonia**	**PZ795339**	**PZ803559**	**PZ803595**	**PZ803575**
***B. imbricatum***	**L-400474 (O)**	**New Caledonia**	**PZ795344**	**PZ803558**		**PZ803574**
***B. imbricatum***	**L-400101 (O)**	**New Caledonia**	**PZ795340**	**PZ803560**	**PZ803596**	**PZ803576**
***B. imbricatum***	**L-400467 (O)**	**New Caledonia**	**PZ795343**	**PZ803561**		**PZ803577**
***B. imbricatum***	**L-401113 (O)**	**New Caledonia**	**PZ795341**	**PZ803562**	**PZ803597**	**PZ803578**
***B. imbricatum***	**L-400655 (O)**	**New Caledonia**	**PZ795342**	**PZ803563**		**PZ803579**
***B. imbricatum***	**Tibell 19905 (UPS)**	**New Caledonia**		**PZ803565**	**PZ803599**	
***B. imshaugii***	**Wedin 4873 (UPS)**	**New Zealand**		**PZ803567**	**PZ803601**	**PZ803586**
***B. insigne***	**Wheeler & Nelson 1014 (S)**	**Chile**				**PZ803585**
***B. insigne***	**Wedin 8682 (S)**	**Argentina**				**PZ803584**
**B. cf. insigne**	**L-401147 (O)**	**New Caledonia**	**PZ795337**	**PZ803556**		**PZ803572**
**B. cf. insigne**	**L-400645 (O)**	**New Caledonia**	**PZ795338**	**PZ803557**	**PZ803594**	**PZ803573**
***B. kinabaluense***	**Paukov 2094 (B)**	**Malaysia**				**PZ803589**
***B. kinabaluense***	**Paukov 2076 (B)**	**Malaysia**				**PZ803590**
*B. melanocarpum*	Benitez, Gonzalez & Prieto 4006 (S)	Ecuador		MF954572	MF954595	MF954538
*B. melanocarpum*	Coca 1703 (FAUC)	Colombia		MF954568	MF954591	MF954536
*B. melanocarpum*	Coca 1806 (FAUC)	Colombia		MF954569	MF954592	MF954550
*B. melanocarpum*	Soto 26 (CUVC)	Colombia		MF954570	MF954593	MF954551
*B. melanocarpum*	Malaver 97 (UDBC)	Colombia		MF954567	MF954590	MF954547
*B. melanocarpum*	Home s.n. (CUVC)	Colombia		MF954571	MF954594	MF954545
*B. melanocarpum*	Benitez & Prieto 4008 (S)	Ecuador		MF954573	MF954596	MF954549
*B. notatum*	Wedin 9188 (S)	New Zealand		MF954574	MF954597	MF954533
*B. ramuliferum*	Wedin 8652 (S)	Argentina		MF954575	MF954598	MF954534
***B. tibellii***	**Wedin 5005 (UPS)**	**New Zealand**				**PZ803583**
***B. tibellii***	**Wedin 9179 (S)**	**New Zealand**				**PZ803582**
*Sphaerophorus globosus*	L-201265 (O)	Norway	MK812327			
*S. fragilis*	L-196043 (O)	Norway	MK812393			

### Morphological and chemical analyses

The collections were studied under dissecting (Nikon SMZ1270i) and compound microscopes (Nikon ECLIPSE Ni-L). The morphological and anatomical measurements presented in this study are expressed as follows: (minimum–) mean - SD – (mean) – mean + SD (–maximum) units. Conidial measurements are given as extreme values only. For the ascospores, the length refers to the longest diameter (i.e., in the case of subglobose ascospores) and does not take into account their ornamentation (i.e., the electron-dense mazaedial material). Ascospore measurements were carried out in water. Morphological terminology and measurements are based on Wedin (1995b).

Additionally, New Caledonian samples were subjected to thin-layer chromatography (TLC) following the protocols of Culberson (1972) and Culberson and Johnson (1982). The analyses were performed on aluminum plates using solvent system C (toluene 170: acetic acid 30). Chromatograms were examined under UV light (254 and 366 nm) and visible light after charring with 10% sulfuric acid. Whenever possible, TLC was conducted on fertile branches (including ascomata), as these typically exhibit the greatest diversity and concentration of compounds (Wedin 1995a). TLC was performed on all sequenced New Caledonian specimens included in the taxonomic study, with at least one fertile branch examined per species. A subset of the specimens chemically analyzed is presented in Suppl. material 3.

### DNA sequencing

Fifty sequences representing three protein-coding markers [β-tubulin: 16, RNA polymerase II largest subunit (*RPB1*): 10, and DNA replication licensing factor (*MCM7*): 24] were generated for the species of interest. These markers were selected based on their use in the only phylogenetic study of the genus *Bunodophoron* to date. Sequences from samples not deposited in O were generated using the same protocol (Soto Medina et al. 2018). For samples deposited in O, DNA was extracted using the Chelex 100 kit (Bio-Rad, Hercules, California, USA) following the protocol described by Ferencova et al. (2017), and gene amplification for these specimens was carried out using the primers bTub-75F-Buno, bTub-803R-Buno, RPB1-Af, RPB1-773R-Sphaero, MCM7-SphaeF, and MCM7-SphaeR (Matheny et al. 2002; Soto Medina et al. 2018). The thermocycling protocol consisted of an initial denaturation step at 95 °C for 5 min, followed by 34–36 cycles of denaturation at 95 °C for 1 min, annealing at 55 °C for 30 s, and extension at 72 °C for 1 min, with a final extension step at 72 °C for 5–8 min. Additionally, 12 sequences of the internal transcribed spacer (ITS) region, widely adopted as a universal DNA barcode marker for fungi (Schoch et al. 2012), were generated for New Caledonian material deposited in O. These sequences were generated using the primers ITS1F (Gardes and Bruns 1993) and ITS4 (White et al. 1990) under the thermocycling conditions described above. PCR reactions were performed using GoTaq® Green Master Mix (Promega, Madison, Wisconsin, USA). Amplicons were purified with ExoStar-IT (GE Healthcare, Buckinghamshire, UK) and subsequently sequenced at Macrogen Europe (Amsterdam, Netherlands).

### Phylogenetic analyses

In addition to the molecular data generated in this study, most of the *Bunodophoron* sequences published to date (Soto Medina et al. 2018) were included in the phylogenetic analyses (Table 1). Sequences for the four markers were aligned separately using MAFFT v. 7.450 (Katoh 2002; Katoh and Standley 2013), as implemented in GUIDANCE v. 2.02 (Landan and Graur 2008; Sela et al. 2015), with the L-INS-i algorithm. Alignments for each locus were visualized in Geneious Prime v. 2025.1.1 (Biomatters Ltd., Auckland, New Zealand). In view of the high GUIDANCE alignment scores and the lack of significantly ambiguous regions, all sites were retained for the subsequent phylogenetic inferences. The ends of each alignment were trimmed to ensure that all sequences were approximately the same length. This resulted in the following alignment lengths: (1) 552 base pairs for the ITS matrix, (2) 726 base pairs for the β-tubulin matrix, (3) 726 base pairs for the *RPB1* matrix, and (4) 573 base pairs for the *MCM7* matrix.

Prior to concatenation, preliminary single-locus phylogenetic trees were constructed for each marker using IQ-TREE v. 2.3.6 (Minh et al. 2020), with 1,000 ultrafast bootstrap replicates (Hoang et al. 2018) and without partitioning. Ultrafast bootstrap values ≥95% were considered to indicate strong branch support. Topological incongruence between the four single-locus trees was assessed using compat.py v. 3.0 (Kauff and Lutzoni 2002) with a 95% bootstrap threshold for detecting conflicts among strongly supported clades. No significant conflicts with bootstrap support ≥95% were detected, allowing the three protein alignments to be concatenated into a single dataset for phylogenetic inference under a partitioning scheme (Suppl. material 1). The ITS alignment was treated separately, as limited data for this marker are available in the genus *Bunodophoron* (Suppl. material 2).

The concatenated protein-coding alignment consisted of 2,025 positions from 48 specimens. Substitution model partitioning and maximum likelihood (ML) analysis were performed using IQ-TREE v. 2.3.6 (Chernomor et al. 2016; Minh et al. 2020). Eleven initial subsets were defined: three codon positions for β-tubulin, *RPB1*, and *MCM7*, as well as the introns of *RPB1* and β-tubulin. IQ-TREE optimized model fits by collapsing candidate subsets and selecting substitution models based on the Bayesian information criterion (BIC), yielding two independently parameterized subsets: K2P+I applied to the first and second codon positions of the three genes and TNe+G4 to the third codon positions and the introns of *RPB1* and β-tubulin. The ML analysis of the three-locus dataset was carried out with 1,000 ultrafast bootstrap replicates. Two specimens of *Bunodophoron agnetae* Wedin were selected as the outgroup based on preliminary analyses using the related genus *Sphaerophorus* as an outgroup, which positioned *B. agnetae* as sister to all other *Bunodophoron* taxa in the dataset. An ITS tree was constructed separately using the same ML approach, without partitioning and using the selected model K2P+I. In this case, specimens of *S. fragilis* (L.) Pers. and *S. globosus* (Huds.) Vain. were used as outgroups (Marthinsen et al. 2019). The resulting ML trees were visualized using the package ggtree v. 4.1.1.004 (Yu et al. 2017; Xu et al. 2022) in R v. 4.4.2.

## Results and discussion

**Figure 1.**
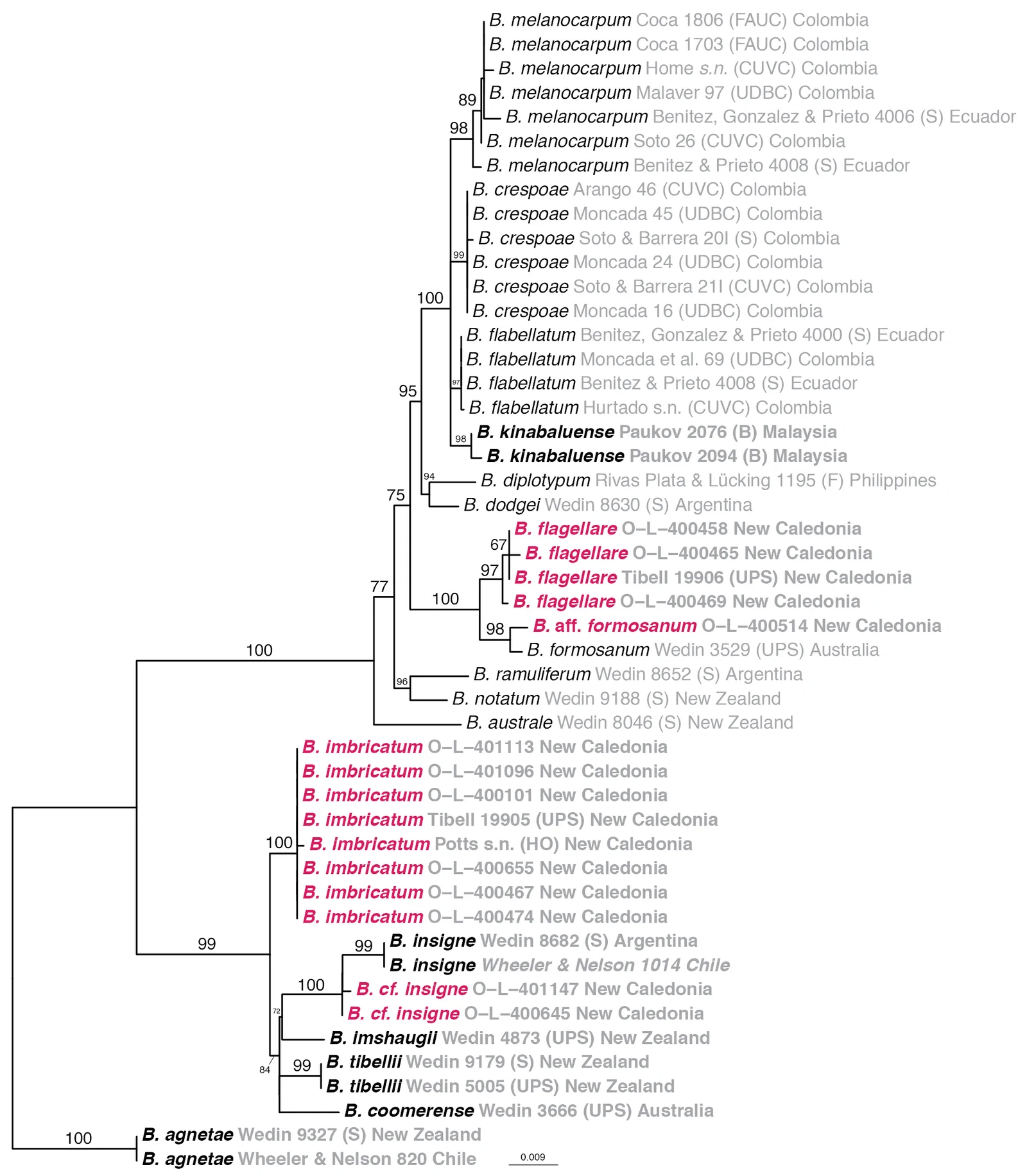
Phylogram representing the best-scoring maximum likelihood tree based on phylogenetic analysis of the genus *Bunodophoron* using three molecular markers (β-tubulin, *MCM7*, and *RPB1*). Values above branches represent bootstrap support values (indicated when greater than or equal to 50). Newly generated sequences are indicated in bold, while others were retrieved from Soto Medina et al. (2018). Tree tips highlighted in red represent samples collected in New Caledonia.

**Figure 2.**
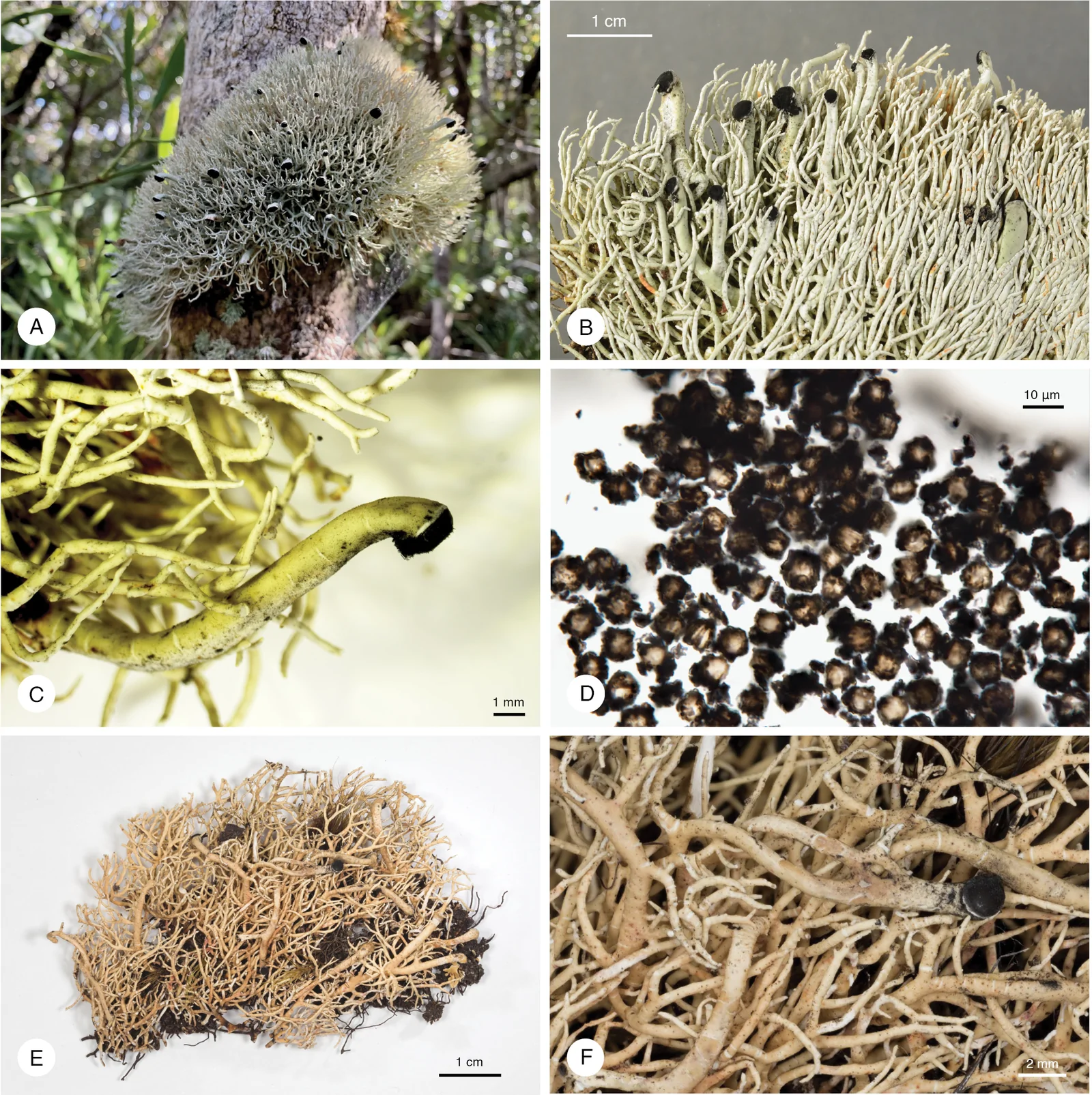
Habit of *Bunodophoron flagellare*, a new species from New Caledonia. **A**. Thallus overview in the field (O-L-400465); **B**. Thallus overview, herbarium specimen (O-L-400465); **C**. Close-up of apothecium (O-L-400465); **D**. Ascospores (O-L-400465); **E**. Holotype: thallus overview (Tibell 19906; UPS); **F**. Holotype: close-up of apothecium (Tibell 19906; UPS).

**Figure 3.**
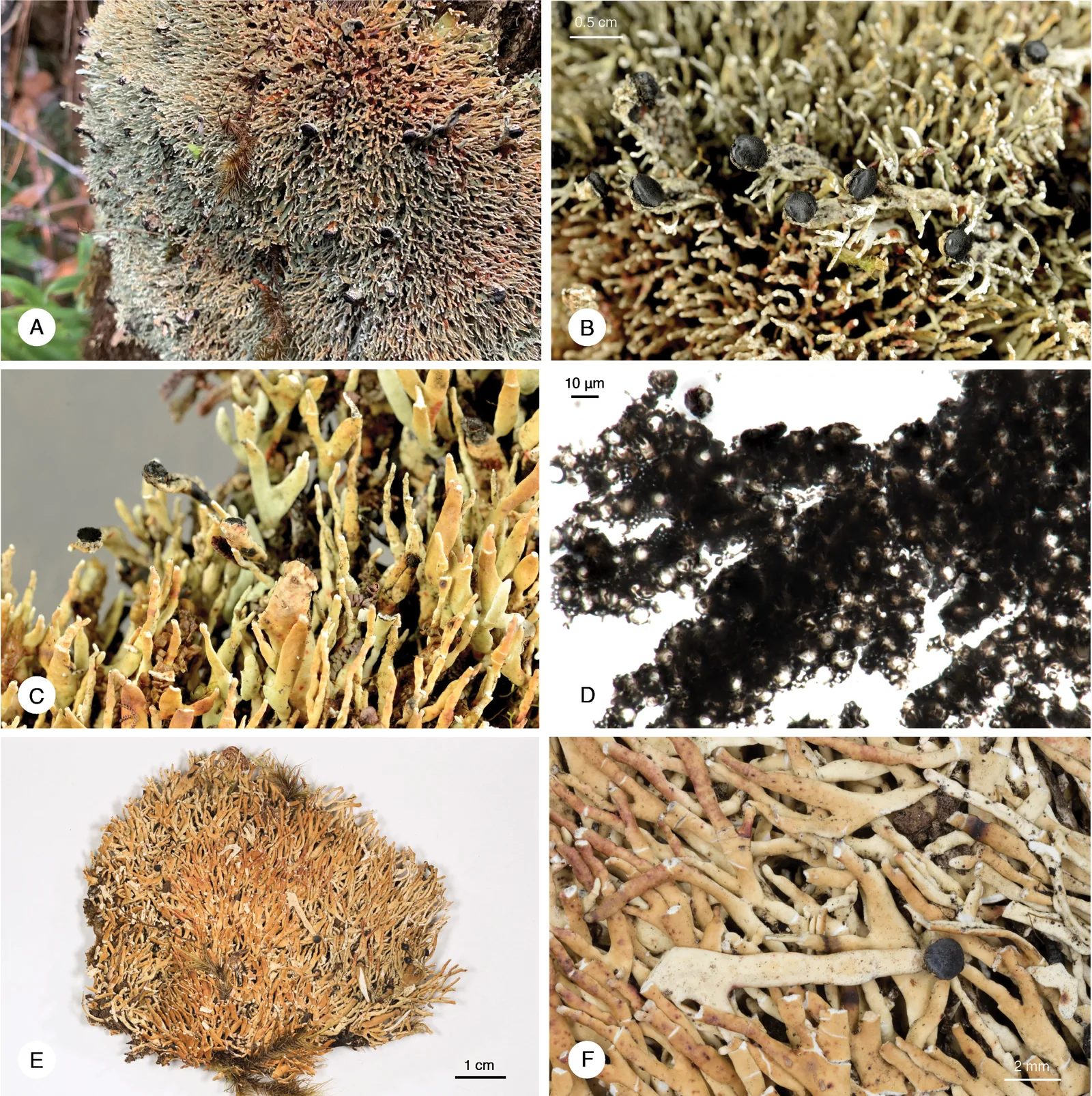
Habit of *Bunodophoron imbricatum*, a new species from New Caledonia. **A**. Thallus overview in the field (O-L-400655); **B, C**. Close-up of fertile branches (**B**. O-L-400655; **C**. O-L-400474); **D**. Ascospores (O-L-401113); **E**. Holotype: thallus overview (Tibell 19905; UPS); **F**. Holotype: close-up of apothecium (Tibell 19905; UPS).

**Figure 4.**
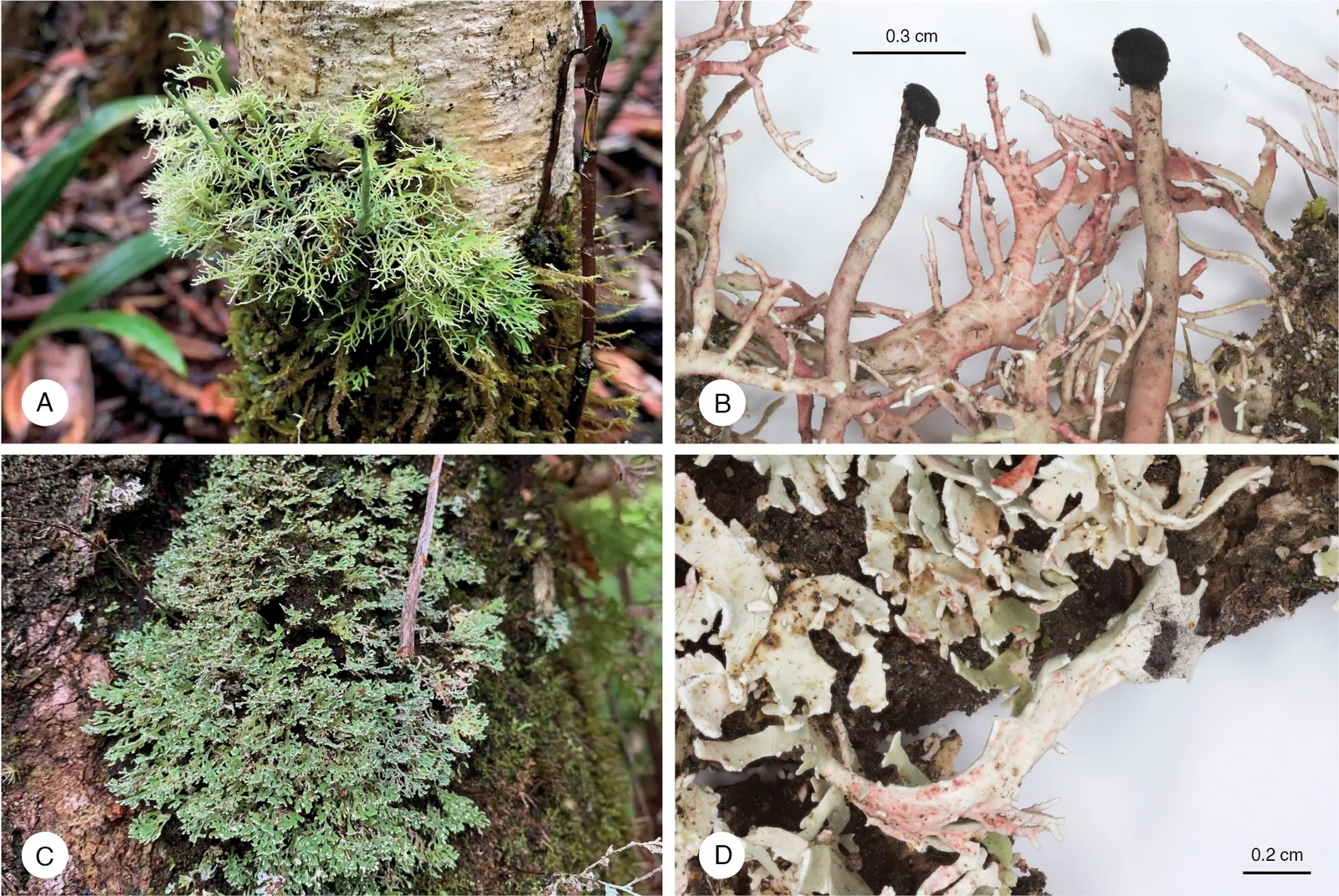
Habit of *Bunodophoron* species from New Caledonia. **A, B**. Bunodophoron cf. formosanum, thallus overview (O-L-400514); **C, D**. Bunodophoron cf. insigne, thallus overview (O-L-400645) and close-up of apothecium (O-L-400508).

Various secondary compounds were identified in New Caledonian material via TLC (Suppl. material 3), including ascomatic acid, 7-*O*-methylascomatic acid, isousnic acid, protocetraric acid, sphaerophorin, stictic acid, and usnic acid. Among these, stictic acid and protocetraric acid are among the most taxonomically significant acetone-soluble metabolites. The best ML three-locus tree (Fig. 1) reveals two strongly supported main clades: one comprising taxa with stictic acid-related compounds (*B. melanocarpum* group; ultrafast bootstrap support [BS] = 100) and the other predominantly including samples with protocetraric acid (*B. insigne* group; BS = 99). The ITS tree (Suppl. material 4), although sparsely populated due to the lack of preexisting data for this locus, agrees topologically with the tree inferred from the concatenated dataset. The topology of the *B. melanocarpum* clade is consistent with the only phylogenetic study on the genus to date (Soto Medina et al. 2018), which focused exclusively on a subset of species containing stictic acid. The New Caledonian taxa can tentatively be grouped into four distinct species-level lineages. Two of these species are newly described here: Bunodophoron flagellare sp. nov. (Fig. 2) and *Bunodophoron imbricatum* sp. nov. (Fig. 3). The remaining New Caledonian taxa are represented by B. cf. formosanum and B. cf. insigne (Fig. 4).

*Bunodophoron flagellare*, resolved within the *B. melanocarpum* clade and composed exclusively of New Caledonian specimens, is potentially endemic to the archipelago. It was recovered as a sister, with strong support (BS = 100), to the lineage encompassing B. cf. formosanum. This lineage also includes one specimen from Australia, identified as *B. formosanum* in Soto Medina et al. (2018). While the two New Caledonian taxa are chemo-morphologically similar, the present assessment (see taxonomic treatment below) suggests that they represent distinct species. This newly described species is also morphologically similar to *B. kinabaluense* in the sense of Ohlsson (1973). However, samples collected near the type locality of *B. kinabaluense*, identified as such and included in the phylogenetic analysis, fall into a separate lineage forming a strongly supported clade (BS = 100) with Neotropical specimens (*B. crespoae*, *B. flabellatum*, and *B. melanocarpum*). It is therefore highly likely that previous reports of *B. kinabaluense* from New Caledonia refer to *Bunodophoron flagellare* or B. cf. formosanum.

*Bunodophoron imbricatum*, resolved within the *B. insigne* clade and composed exclusively of New Caledonian specimens, is likewise potentially endemic to the archipelago. Superficially, it is morphologically similar to *B. imshaugii*, a species apparently widespread in the Southern Hemisphere and previously reported from New Caledonia. However, phylogenetic analyses confirm that the two taxa are well distinguished (see taxonomic treatment below). Additionally, within the *B. insigne* clade, New Caledonian specimens referred to here as B. cf. insigne form a strongly supported clade (BS = 98) with South American material assigned to the same species. However, the relatively long branch supporting this clade suggests that the New Caledonian specimens might represent a distinct, as-yet-undescribed taxon.

These findings underscore the taxonomic richness of New Caledonia, as even a visually striking and conspicuous genus like *Bunodophoron* continues to yield novel species. In total, the presence of four species within the genus in the region is confirmed.

### Taxonomy

#### Bunodophoron flagellare

Taxon classificationFungiLecanoralesSphaerophoraceae

Ant.Simon, N.Borg & Wedin sp. nov.

D0337D9A-DFF5-5B0C-A2C0-752A92A78B41

864872

Fig. 2

##### Type.

New Caledonia, • just south of the summit of Mt. Ouin, in mixed *Araucaria*-hardwood forest, in wet montane area, on trunk of *Cyathea*, alt. 1,050 m, 22°01'S, 166°27'E, 1993, L. Tibell 19906 (holotype: UPS, L-1216169; isotypes PC, NOU; planned to be distributed in Tibell: Caliciales Exsiccata).

##### Diagnosis.

A medium-to-large fruticose species, sparingly branched and characterized by whitish, subterete, long, and slender branches, particularly the lower-order branches. It is morphologically similar to *Bunodophoron formosanum* but differs from it by its less divided branches and the absence of isidioid outgrowths on the upper surface.

##### Etymology.

The epithet refers to the slender, elongated, and whiplash-like aspect of the lower-order branches.

##### Description.

Thallus epiphytic, forming extensive colonies with dense and imbricate patches of elongated and delicate branches. Fertile branches subterete to narrowly compressed, slender, sparingly branched, (11–)15–(21)–26(–32) mm long (*n* = 28) and (0.9–)1.1–(1.5)–1.9(–25) mm wide (*n* = 28); supporting branches slender, narrowly compressed to subterete, usually slightly bent upwards. Branches of lower orders formed near the base of the thalli, subterete, slender to flagelliform, sparingly branched or occasionally simple. Upper surface white-grey to nearly white, with infrequent patches of yellow-orange tint, smooth, without pruina. Lower surface, similarly colored, smooth. Ascomata sparse to abundant, terminal, (0.8–)1.3–(1.9)–2.5(–3.0) mm wide (*n* = 27). Mazaedia subapically exposed, conspicuous, strongly sooting. Ascospores (5.0–)6.2–(7.2)–8.2(–9.7) μm diam. (*n* = 77), light brown to dark grey, with dark ornamentation. Pycnidia sparse to abundant, in the tips of ultimate branches. Conidia bacilliform, 4–5.5 × 1–1.5 µm (*n* = 45).

##### Chemistry.

Sphaerophorin, stictic and constictic acids, unidentified compound (accessory). Medulla pale K+, Pd– or Pd+ orange.

##### Additional specimens examined.

Additional specimens examined. **New Caledonia**: • South Province, Païta, along trail towards Mont Mou, Réserve Spéciale du Mont Mou, 22°03.713'S, 166°20.749'E, alt. 1,146 m, epiphytic, in high-elevation, shrubby maquis transitioning into rainforest, 4 November 2022, A. Simon 838 (O; L-400255); • Païta, Réserve Spécial Botanique du Mont Humboldt, along the trail, 21°52.975'S, 166°23.948'E, alt. 1,265 m, epiphytic in high-elevation rainforest, 27 November 2022, A. Simon 1041 (O; L-400458) with R. Amice, A. Evankow, R. Haugan, C. Laudereau & E. Timdal; • *ibid*., 21°53.002'S, 166°24.758'E, alt. 1330 m, epiphytic, in fairly dry high-elevation rainforest, A. Simon 1048 (O; L-400465) with R. Amice, A. Evankow, R. Haugan, C. Laudereau & E. Timdal; • *ibid*., 21°52.996'S, 166°24.766'E, alt. 1,335 m, 27 November 2022, A. Simon 1050 (O; L-400469) with R. Amice, A. Evankow, R. Haugan, C. Laudereau & E. Timdal; • Yaté Commune, Piste des Dzumacs, along the trail towards Mont Ouin, at the summit, –22.0163, 166.4726, alt. 1,320 m, epiphytic in high-altitude rainforest, on exposed ridge, 30 November 2025, A. Simon 2334 (S; NOU), with R. Amice; • *ibid*., approximately 100 m S of the summit, –22.0173, 166.4724, alt. 1,280 m, epiphytic in high-altitude rainforest, 30 November 2025, A. Simon 2423 (S; NOU), with R. Amice; • *ibid*., approximately 200 m S of the summit, –22.0181, 166.4727, alt. 1,260 m, epiphytic in high-altitude rainforest, 30 November 2025, A. Simon 2445 (S; NOU), with R. Amice; • *ibid*., at the summit, –22.0162, 166.4728, alt. 1,317 m, epiphytic in high-altitude rainforest, on exposed ridge, 2025 November 30, A. Simon 2449 (S; NOU), with R. Amice.

##### Habitat and distribution.

This newly described species is currently known only from New Caledonia, where it may be endemic. It is found exclusively in mountain rainforests or very moist maquis vegetation (shrubland) transitioning into forest at high altitudes (>1,000 m). It appears to favor semi-open habitats with relatively high light availability.

##### Notes.

**Figure 5.**
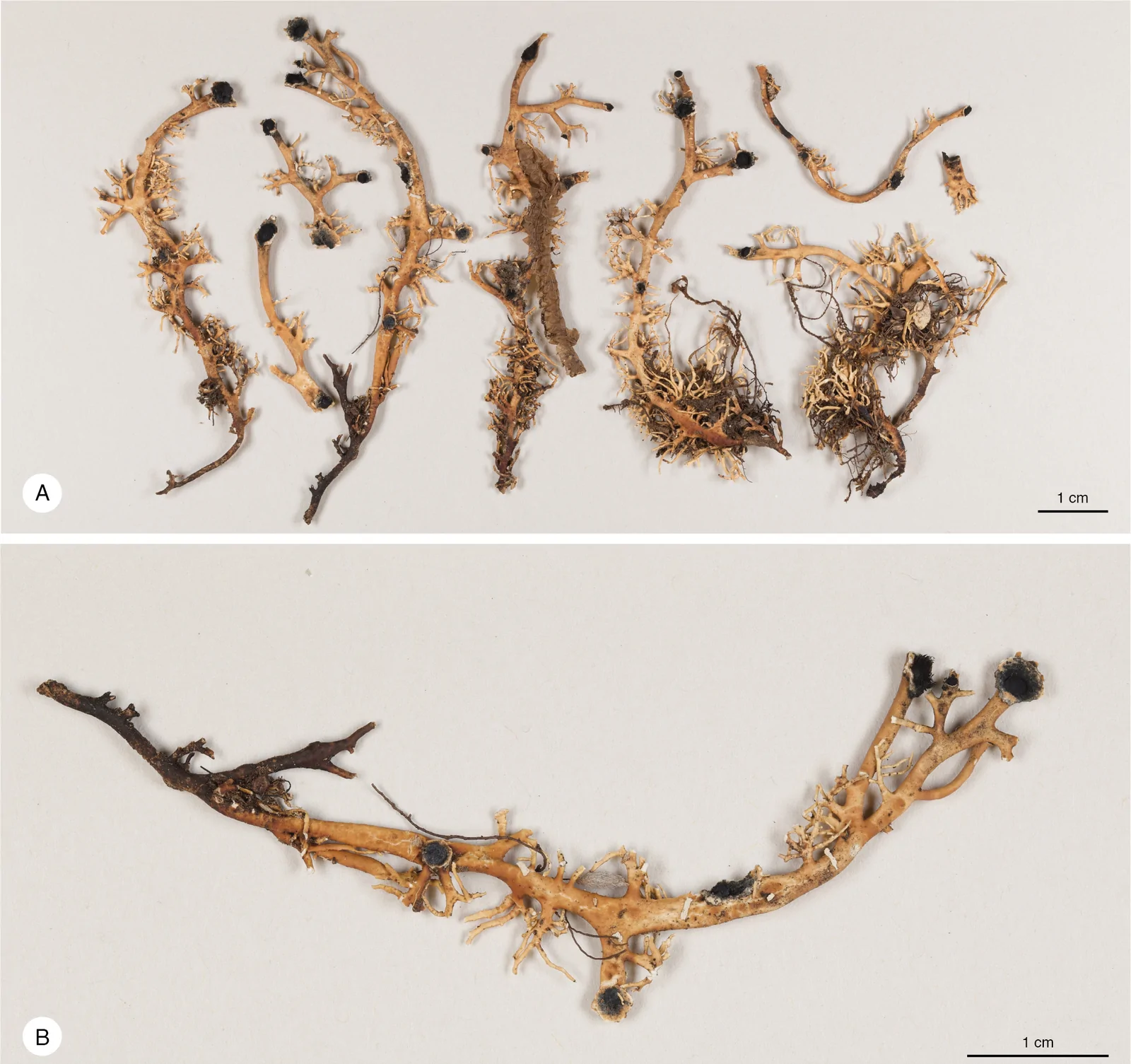
Isotype of *Bunodophoron kinabaluense* (M.E. Hale 28656, US). **A**. Thallus overview; **B**. Close-up of a fertile branch with laminal apothecia. Photographs: Jennifer Kearey.

This species may have previously been confused with *B. kinabaluense*, which was originally described from Borneo (as *Pseudosphaerophorus kinabaluensis* Satô; Satô 1967) and later reported from New Caledonia (Ohlsson 1973). *Pseudosphaerophorus* Satô was characterized by laminal apothecia and two-celled ascospores. The ascospore observations were shown by Ohlsson to be erroneous. Ohlsson’s concept of this species was mainly based on material of *B. flagellare* and on material of *B. formosanum* s. lat., and Ohlsson commented that the type material was very atypical for the species. The phylogenetic analyses revealed that they are only distantly related: samples morphologically similar to the type of *B. kinabaluense* and collected near the type locality of this species were recovered in a distinct clade. The isotype of *B. kinabaluense* (M.E. Hale 28656; US) was examined. This specimen differs from the material collected in New Caledonia, particularly in having main branches that extend from the margins of old apothecia with repeated production of new fruiting bodies, giving the impression of laminally produced apothecia (Fig. 5). The branching pattern along the fertile branches is also very different. The New Caledonian material is therefore not considered conspecific with this species.

#### Bunodophoron formosanum

Taxon classificationFungiLecanoralesSphaerophoraceae

(Zahlbr.) Wedin, Pl. Syst. Evol. 187(1–4): 233 (1993)

5820F89F-572E-5B9E-B259-2854D4BF1B1F

361306

Fig. 4A, B

Sphaerophorus melanocarpus subsp. formosanus Zahlbr., Feddes Repert. Spec. Nov. Regni veg. 31: 206 (1933). Basionym.
Sphaerophorus formosanus (Zahlbr.) Asahina, J. Jap. Bot.: 667 (1938). Synonym.

##### Type.

Taiwan, • Raisha, 1926, Asahina F 274 (holotype: W!; isotype: TNS, not seen).

##### Description.

See Wedin (1995b) for a detailed description.

##### Chemistry.

Sphaerophorin and the stictic acid complex (including constictic and cryptostictic acids) were reported by Wedin (1995a) and are present in the New Caledonian material examined and referred to as B. cf. formosanum.

##### Specimens examined.

**New Caledonia**: • South Province, Boulouparis, Réserve de Faune et de Flore du Mont Do, along the road near the peak, 21°45.471'S, 166°00.062'E, 960 m, epiphytic, in high-elevation rainforest, 2 December 2022, A. Simon 1097 (O; L-400514) with A. Evankow, R. Haugan, E. Möller & E. Timdal; • Yaté Commune, Piste des Dzumacs, along the trail towards Mont Ouin, at the summit, –22.0279, 166.4705, alt. 930 m, epiphytic in high-altitude rainforest, on exposed ridge, 30 November 2025, A. Simon 2421 (S; NOU), with R. Amice; • Pied de la Table Unio, July 1909, Le Rat (UPS; L-68955).

##### Habitat and distribution.

This species is found in tropical and subtropical high-altitude rainforests and is thought to be widely distributed across the Paleotropics and in Australasia. It has previously been reported from New Caledonia (Tibell 1987; Aptroot and John 2015). The few New Caledonian samples tentatively identified as B. cf. formosanum are from mountain rainforests at moderate altitudes (<1,000 m).

##### Notes.

As noted by Wedin (1995b), *B. formosanum*, as currently circumscribed, exhibits considerable morphological variation, and it is possible that multiple taxa are subsumed under this name. The presence of stictic acid and isidioid branchlets has been considered diagnostic, although their abundance has been shown to vary substantially across the species’ range. These features are present in the New Caledonian material provisionally identified as B. cf. formosanum; however, it remains unclear whether this material belongs to that taxon *sensu stricto*. In the phylogenetic analysis, the only New Caledonian specimen sequenced was recovered as sister to an Australian specimen identified as *B. formosanum* (Soto Medina et al. 2018). Given that no material from Taiwan, the type locality of the species, has been sequenced to date, the taxonomic status of the New Caledonian material remains tentative.

#### Bunodophoron imbricatum

Taxon classificationFungiLecanoralesSphaerophoraceae

Ant.Simon, N.Borg & Wedin sp. nov.

5B0F9A14-E246-5D5A-B77D-8D9E049CD58E

864873

Fig. 3

##### Etymology.

The epithet refers to the slender and elongated aspect of the main branches.

##### Type.

New Caledonia, • just south of the summit of Mt. Ouin, in mixed *Araucaria*-hardwood forest, in wet montane area, on trunk of *Cyathea*, Alt. 1,050 m, 22°01'S, 166°27'E, 1993, L. Tibell 19905 (holotype: UPS, L-1216171; isotypes PC, NOU; planned to be distributed in Tibell: Caliciales Exsiccata).

##### Diagnosis.

A fruticose species of variable size, with scarcely divided, flattened branches and a brownish-ochre upper surface. It is morphologically similar to *Bunodophoron agnetae* but differs from it in its larger size and the absence of isosphaeric acid. *Bunodophoron imshaugii* is larger and has more distinctly reddish-brown ascospores.

##### Description.

Thallus epiphytic, forming compact and usually extensive patches of imbricate, mostly decumbent, sparingly divided or simple branches. Fertile branches generally broadly flattened, narrowly compressed in apical parts, simple or sparingly branched, (11–)12–(15)–18(–24) mm long and (1.5–)1.6–(2.3)–3.1(–4.9) mm wide (*n* = 26). Sterile branches broadly flattened to less rarely subterete, simple to sparingly branched. Upper surface pale brown to light orange in the basal part of the branches, smooth, without pruina. Lower surface lighter in color than the upper surface, whitish with occasional light orange tint, irregular, strongly wrinkled to seemingly scrobiculate. Ascomata abundant to sparse, hemispherical to globose, terminal, (0.9–)1.2–(1.7)–2.3(–2.5) mm wide (*n* = 19). Mazaedia subapically exposed, dense, inconspicuous. Ascospores (4.7–)7.0–(9.0)–11.0(–12.6) μm diam. (*n* = 77), hyaline to light grey, strongly hydrophobic, with black ornamentation. Pycnidia sparse, in apices of branches. Conidia bacilliform, 3.2–4.1 × 1.5 µm.

##### Chemistry.

Ascomatic acid, 7-*O*-methylnorascomatic acid (trace), isousnic acid (accessory), sphaerophorin, and protocetraric acid. In one of the specimens examined (O-L-400101), protocetraric acid is absent (Suppl. material 3). Medulla K–, Pd–, or Pd+ orange.

##### Specimens examined.

Additional specimens examined. **New Caledonia**: • North Province, Hienghène, the trail to Les roches de la Ouaième, 20°38.555'S, 164°51.627'E, 950 m, on tree trunk, in high-elevation rainforest, 8 December 2022, A. Evankow (O; L-400101) with R. Haugan, E. Möller, A. Simon & E. Timdal; • South Province, Païta, Réserve Spécial Botanique du Mont Humboldt, Refuge du Humboldt, 21°53.001'S, 166°24.763'E, 1,340 m, epiphytic, in high-elevation rainforest, 27 November 2022 A. Simon 1238 (O; L-400655) with R. Amice, A. Evankow, C. Laudereau, R. Haugan & E. Timdal; • *ibid*., between Refuge du Humboldt and the peak, 21°52.996'S, 166°24.766'E, 1,335 m, epiphytic, in fairly dry high-elevation rainforest, A. Simon 1050 (O; L-400467) with R. Amice, A. Evankow, R. Haugan, C. Laudereau & E. Timdal; • *ibid*., along the trail, 21°52.888'S, 166°25.047'E, 1420 m, epiphytic in high-elevation maquis, 28 November 2022, A. Simon 1057 (O; L-400474) with R. Amice, A. Evankow, R. Haugan, C. Laudereau & E. Timdal; • *ibid*., 21°52.829'S, 166°24.545'E, 1390 m, on tree trunk, 27 November 2022, R. Haugan (O; L-401096), R. Amice, A. Evankow, C. Laudereau, A. Simon & E. Timdal; • *ibid*., Thio, Réserve Spécial Botanique du Mont Humboldt, Refuge du Humboldt, 21°52.897'S, 166°24.793'E, 1340 m, epiphytic, in high-elevation rainforest, 28 November 2022, R. Haugan (O; L-401113) with R. Amice, A. Evankow, C. Laudereau, A. Simon & E. Timdal; • Païta, Piste des Dzumac, départ du sentier du Mont Ouin, forêt de nuage, –22.02814, 166.47032, E. Lebreton 1881 (LG); • Païta, along trail towards Mont Mou, Réserve Spéciale du Mont Mou, close to the summit, –22.0617, 166.348, alt. 1,150 m, epiphytic within high-altitude rainforest, 10 November 2025, A. Simon 2332 (S; NOU), with J. Kearey & T. Lindberg; • *ibid*., –22.0618, 166.3451, alt. 1,150 m, epiphytic, on the ridge crest, close to the summit, 10 November 2025, A. Simon 2339 (S; NOU), with J. Kearey & T. Lindberg; • Piste des Dzumacs, at the beginning of the trail towards Mont Ouin, –22.0281, 166.4704, alt. 930 m, epiphytic in high-altitude rainforest, 30 November 2025, A. Simon 2457 (S; NOU), with R. Amice; • Mt Koghi track, 22°11'S. 166°32'E, 900 m, epiphytic, 21 February 1991, B.M. Potts *s.n*. (HO; 516529).

##### Habitat and distribution.

*Bunodophoron imbricatum* is currently only known from New Caledonia, occurring in mountain rainforests and high-elevation maquis vegetation. Like *B. flagellare*, this species appears to favor semi-open habitats with relatively high light availability. The two newly described species seem to share highly similar ecological niches and frequently occur sympatrically.

##### Notes.

This species is morphologically most similar to *Bunodophoron agnetae* but differs in both thallus architecture and chemistry. *Bunodophoron imbricatum* forms extensive patches of imbricate branches, whereas *B. agnetae* lacks this growth form and produces isosphaeric acid, along with smaller, nearly hyaline ascospores. Despite its morphological similarity to *B. agnetae*, phylogenetic analyses place *B. imbricatum* with protocetraric-acid-containing species (Fig. 1). Within this group, it may bear a superficial resemblance to the cool-temperate *B. imshaugii* and the tropical *B. coomerense*, but neither of these species develops extensive imbricate patches. The comparatively small size of *B. imbricatum* likely contributes to these superficial similarities; however, molecular evidence clearly supports the New Caledonian material as representing a distinct species.

#### Bunodophoron insigne

Taxon classificationFungiLecanoralesSphaerophoraceae

(Laurer) Wedin, Pl. Syst. Evol. 187(1–4): 233 (1993)

581959F9-699B-591D-9AE8-AA33D61406E8

361308

Fig. 4C, D

Sphaerophorus insignis Laurer [as ‘Sphaerophoron insigne’], Linnaea 2: 45 (1827). Basionym.

##### Type.

Australia, • Sieber s. n. [lectotype: BM!, designated by Ohlsson in Tibell (1987)]. Australia, • Victoria, junction of Coast Range Road and Cabon Road, Errinundra Plateau, 18.5 Ian SSE of Bendoc, 37°18', 148°51'E, 980 m, 1986, Elix 19998 and Streimann (Elix, Lich. Australasici Exs. 220) [epitype: CANB!, designated by Wedin (1995b)].

##### Description.

See Wedin (1995b) for a detailed description.

##### Chemistry.

Protocetraric acid, ascomatic acid, 7-*O*-methylascomatic acid, and sphaerophorin. The New Caledonian material seemingly lacks ascomatic and 7-*O*-methylascomatic acids.

##### Specimens examined.

**New Caledonia**: • South Province, Boulouparis, Réserve de Faune et de Flore du Mont Do, along the road near the peak, 21°45.455'S, 166°0.044'E, 940 m, epiphytic, 2 December 2022, R. Haugan (O; L-401147) with A. Evankow, E. Möller, A. Simon & E. Timdal; • Réserve de Faune et de Flore du Mont Do, just N of the peak, 21°45.196'S, 165°59.996'E, 1,017 m, epiphytic, in high-elevation rainforest, 2 December 2022, A. Simon 1091 (O; L-400508) with A. Evankow, R. Haugan, E. Möller & E. Timdal; • Sarraméa, along trail from Sarraméa to Plateau de Dogny, 21°37.257'S, 165°52.490'E, 910 m, epiphytic, in mid-elevation rainforest, 13 December 2022, A. Simon 1228 (O; L-400645) with A. Evankow, R. Haugan, E. Möller & E. Timdal.

##### Habitat and distribution.

*Bunodophoron insigne* is a common, shade-tolerant species primarily known from temperate rainforests in Australasia and southern South America (Wedin 1995a). It is reported here for the first time from New Caledonia, based on specimens collected at two localities in humid mountain rainforests.

##### Notes.

This species is highly variable, and its taxonomy is likely not well understood (Wedin 1992). The presence of a thalline receptacle and the phylogenetic placement of the New Caledonian material identified as such suggest that it may correspond to this species. However, the genetic divergence between the two sequenced samples and the South American material raises the possibility that these may represent two distinct taxa.

### Taxa not confirmed from New Caledonia

The following taxa were reported from New Caledonia but could not be confirmed in the present study.

#### Bunodophoron australe

Taxon classificationFungiLecanoralesSphaerophoraceae

(Laurer) A.Massal., Mem. Reale Ist. Veneto Sci. 10: 76 (1861)

E366403F-FB4E-58AC-B619-496CA9AFA2C9

≡ Sphaerophorus australis Laurer [as ‘Sphaerophoron australe’], Linnaea 2: 44 (1827).

##### Type.

Australia, on bark, Sieber s. n. [BM!, lectotype designated by Ohlsson in Tibell (1987); M!, PC, isolectotypes].

##### Notes.

*Bunodophoron australe* is widely distributed in the cool-temperate forests of the Southern Hemisphere. Aptroot and John (2015) reported it from New Caledonia as Bunodophoron cf. australe (Laurer) A.Massal., based on a single specimen (H. Hürlimann no. 4148; M; not seen). In the present study, the presence of this species could not be confirmed.

#### Bunodophoron imshaugii

Taxon classificationFungiLecanoralesSphaerophoraceae

(Ohlsson) Wedin, Pl. Syst. Evol. 187(1 –4): 233 (1993)

C3A8C176-4717-55BF-A248-F7B09D037C76

≡ Sphaerophorus imshaugii Ohlsson, in Galloway, New Zealand J. Bot. 21(2): 197 (1983) = Sphaerophorus australis var. *proliferus* F. Wilson [as ‘*Sphaerophoron australe* var. *australeprolifera*’], J. Linn. Soc., Bot. 28: 370 (1891).

##### Type.

Argentina, Isla de los Estados, Bahia Primera, on W side of lake behind inner bay, sea level, Imshaug 52368 (holotype: MSC!).

##### Notes.

Although this species was reported from New Caledonia (Elix and McCarthy 2008), its presence in the archipelago could not be confirmed, and it is possible that this identification was based on material referable to the taxon described here as *B. imbricatum*.

#### Bunodophoron kinabaluense

Taxon classificationFungiLecanoralesSphaerophoraceae

(M. Satô) Wedin, Pl. Syst. Evol. 187(1–4): 233 (1993)

7B772A08-2F14-5A80-A283-2762D9CF2009

≡ Pseudosphaerophorus kinabaluensis M. Satô, Miscell. bryol. lichenol., Nichinan 4(7): 108 (1968) [1967] ≡ Sphaerophorus kinabaluensis (M. Satô) Ohlsson, in Tibell, Symb. bot. upsal. 27(no. 1): 228 (1987).

##### Type.

Borneo. Kinabalu Nat. Park, Hale 28656 (holotype in Herb. Univ. Ibaraki, Japan, not seen; US!, isotype).

##### Notes.

This species was reported from New Caledonia by Ohlsson (1973) but most likely corresponds to *B. flagellare* or *B. formosanum* s. lat. in this region, as discussed above.

#### Bunodophoron ramuliferum

Taxon classificationFungiLecanoralesSphaerophoraceae

(I. M. Lamb) Wedin, Pl. Syst. Evol. 187(1–4): 234 (1993)

97D8CBC8-5E8B-5A0D-BBAD-A2BE8209F35B

≡ Sphaerophorus ramulifer I.M. Lamb, Farlowia 4(4): 426 (1955).

##### Type.

Argentina, Patagonia, Lago Frias, on trunk of *Fitzroya* near the lake, 1950, I. Mackenzie Lamb 5977 (UPS! L-108377, isotype).

##### Notes.

This species was reported from New Caledonia by Tibell (1987), based on a single specimen (Bonati *s.n*.; UPS), which could not be located. The presence of this species in New Caledonia could not be confirmed.

## Supplementary Material

XML Treatment for Bunodophoron flagellare

XML Treatment for Bunodophoron formosanum

XML Treatment for Bunodophoron imbricatum

XML Treatment for Bunodophoron insigne

XML Treatment for Bunodophoron australe

XML Treatment for Bunodophoron imshaugii

XML Treatment for Bunodophoron kinabaluense

XML Treatment for Bunodophoron ramuliferum

## Additional information

### Artificial Intelligence (AI) use

The authors accept full responsibility for the content of the manuscript, including the disclosure of any use of AI.

No AI tools were used in the preparation of this manuscript.

### Author ORCIDs

N.H. Borg https://orcid.org/0009-0005-7659-5615

M. Wedin https://orcid.org/0000-0002-8295-5198

E. Timdal https://orcid.org/0000-0003-4524-0617

M. Prieto https://orcid.org/0000-0002-1692-9821

A. Simon https://orcid.org/0000-0002-7766-5751

### Data availability

All of the data that support the findings of this study are available in the main text or Supplementary Information.

## Supplementary materials

Supplementary material 1 Concatenated three-locus alignment of *Bunodophoron sequences* (β-tubulin, MCM7, and RPB1) Data type nex Explanation note Sequence headers indicate taxon names and correspond either to collector and collection numbers or to herbarium accession numbers (for specimens deposited in O). A NEXUS partition block is provided to delimit the positions of the three loci. https://binary.pensoft.net/file/1770363This dataset is made available under the Open Database License (http://opendatacommons.org/licenses/odbl/1.0/). The Open Database License (ODbL) is a license agreement intended to allow users to freely share, modify, and use this Dataset while maintaining this same freedom for others, provided that the original source and author(s) are credited. Nora Helene Borg, Mats Wedin, Einar Timdal, María Prieto, Antoine Simon

Supplementary material 2 Alignment of *Bunodophoron*ITS sequences Data type nex Explanation note Headers refer to GenBank accession numbers. Sequence headers indicate taxon names and herbarium accession numbers (O). https://binary.pensoft.net/file/1770364This dataset is made available under the Open Database License (http://opendatacommons.org/licenses/odbl/1.0/). The Open Database License (ODbL) is a license agreement intended to allow users to freely share, modify, and use this Dataset while maintaining this same freedom for others, provided that the original source and author(s) are credited. Nora Helene Borg, Mats Wedin, Einar Timdal, María Prieto, Antoine Simon

Supplementary material 3 Thin-layer chromatography (TLC) plate displaying 10 selected *Bunodophoron* specimens from New Caledonia alongside a control sample Data type pdf Explanation note The TLC plate was developed in solvent C (toluene 170: acetic acid 30) and photographed immediately after charring with 10% sulfuric acid. The upper edge of the image marks the migration front. The image has been cropped to show only samples relevant to the study. Identified substances are noted for representative samples. https://binary.pensoft.net/file/1770365This dataset is made available under the Open Database License (http://opendatacommons.org/licenses/odbl/1.0/). The Open Database License (ODbL) is a license agreement intended to allow users to freely share, modify, and use this Dataset while maintaining this same freedom for others, provided that the original source and author(s) are credited. Nora Helene Borg, Mats Wedin, Einar Timdal, María Prieto, Antoine Simon

Supplementary material 4 Best-scoring maximum likelihood tree based on phylogenetic analysis of the genus *Bunodophoron* using the nuclear ribosomal internal transcribed spacer region (ITS) Data type pdf Explanation note Numbers above branches represent bootstrap support values (indicated when greater than or equal to 50). https://binary.pensoft.net/file/1770366This dataset is made available under the Open Database License (http://opendatacommons.org/licenses/odbl/1.0/). The Open Database License (ODbL) is a license agreement intended to allow users to freely share, modify, and use this Dataset while maintaining this same freedom for others, provided that the original source and author(s) are credited. Nora Helene Borg, Mats Wedin, Einar Timdal, María Prieto, Antoine Simon
