# Supplementary material for: Taxonomy and phylogeny of the lichen genus Bunodophoron (Sphaerophoraceae, Lecanorales) in New Caledonia, with the description of two new species
Source: MycoKeys. 2026 Sep 11;140:79–101. doi: 10.3897/mycokeys.140.197771 (PMC13589296; doi:10.3897/mycokeys.140.197771)
Supplement: Supplementary file 1 [file mycokeys-140-079-s003.pdf]

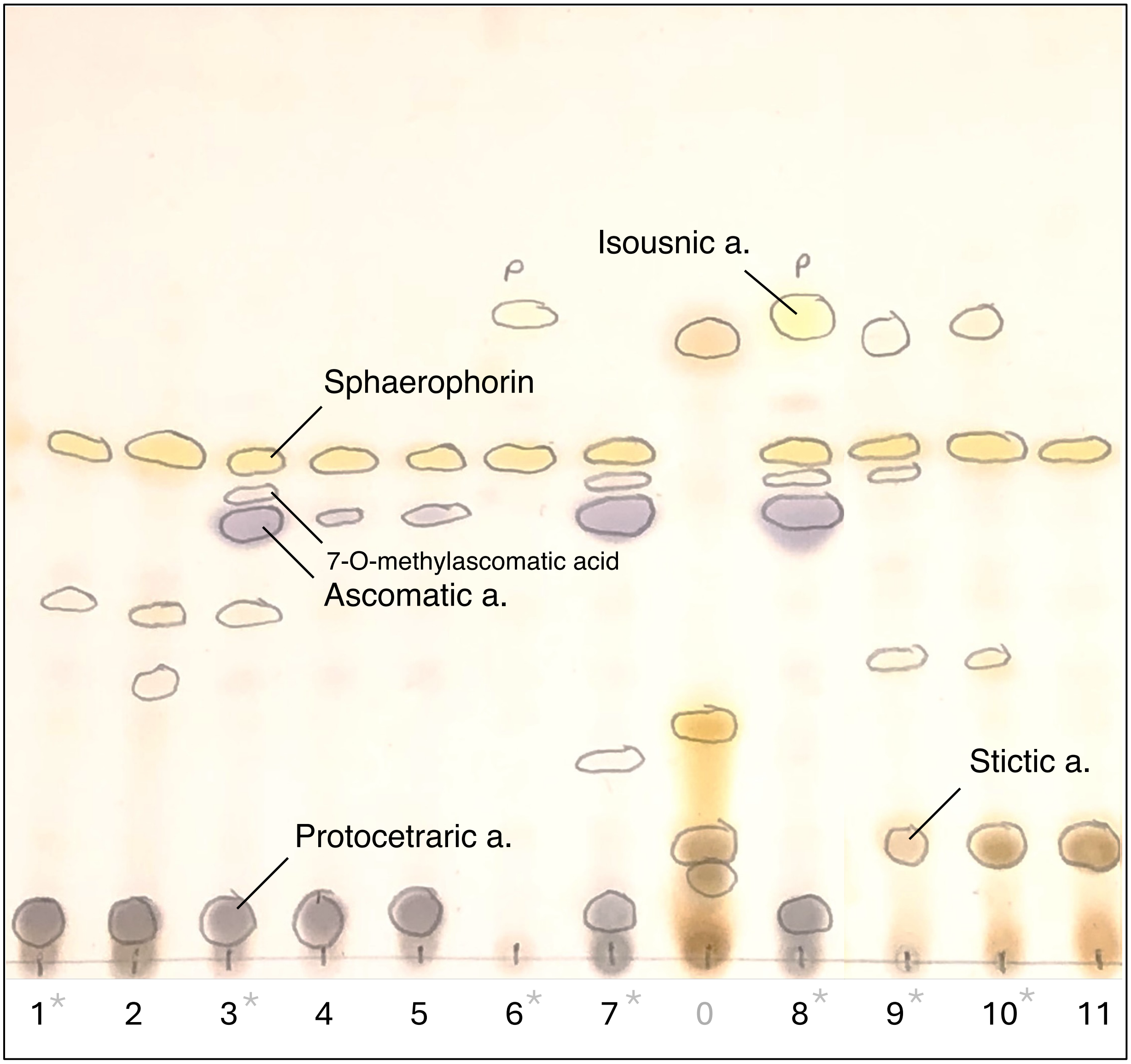

Figure 1. Thin-layer chromatography (TLC) plate displaying 10 selected *Bunodophoron* specimens from New Caledonia alongside a control sample. The TLC plate was developed in solvent C (toluene 170: acetic acid 30) and photographed immediately post-charring with 10% sulfuric acid. The upper edge of the image marks the migration front. The image has been cropped to show only samples relevant to the study. Identified substances are noted for representative samples. The control spot (0) includes the following substances atranorin (*Parmeotrema crinitum*), norstictic acid (*Pleurosticta acetabulum*) and stictic acid (*Parmotrema crinitum*). Sample identifications are as follows: **1–2**: *B. insigne* (O-L-401147, O-L-400645); **3–8**: *B. imbricatum* (O-L-400474, O-L-401096, O-L-400467, O-L-400101, O-L-401113, O-L-400655); **9–11** : *B. flagellare* (O-L-400458; O-L-400465; O-L-400469). Asterisks denote samples for which TLC was performed on fertile branches (including ascomata). Abbreviation: a = acid; P = pigment.
