# Supplementary material for: Taxonomy and phylogeny of the lichen genus Bunodophoron (Sphaerophoraceae, Lecanorales) in New Caledonia, with the description of two new species
Source: MycoKeys. 2026 Sep 11;140:79–101. doi: 10.3897/mycokeys.140.197771 (PMC13589296; doi:10.3897/mycokeys.140.197771)
Supplement: Supplementary file 2 [file mycokeys-140-079-s004.pdf]

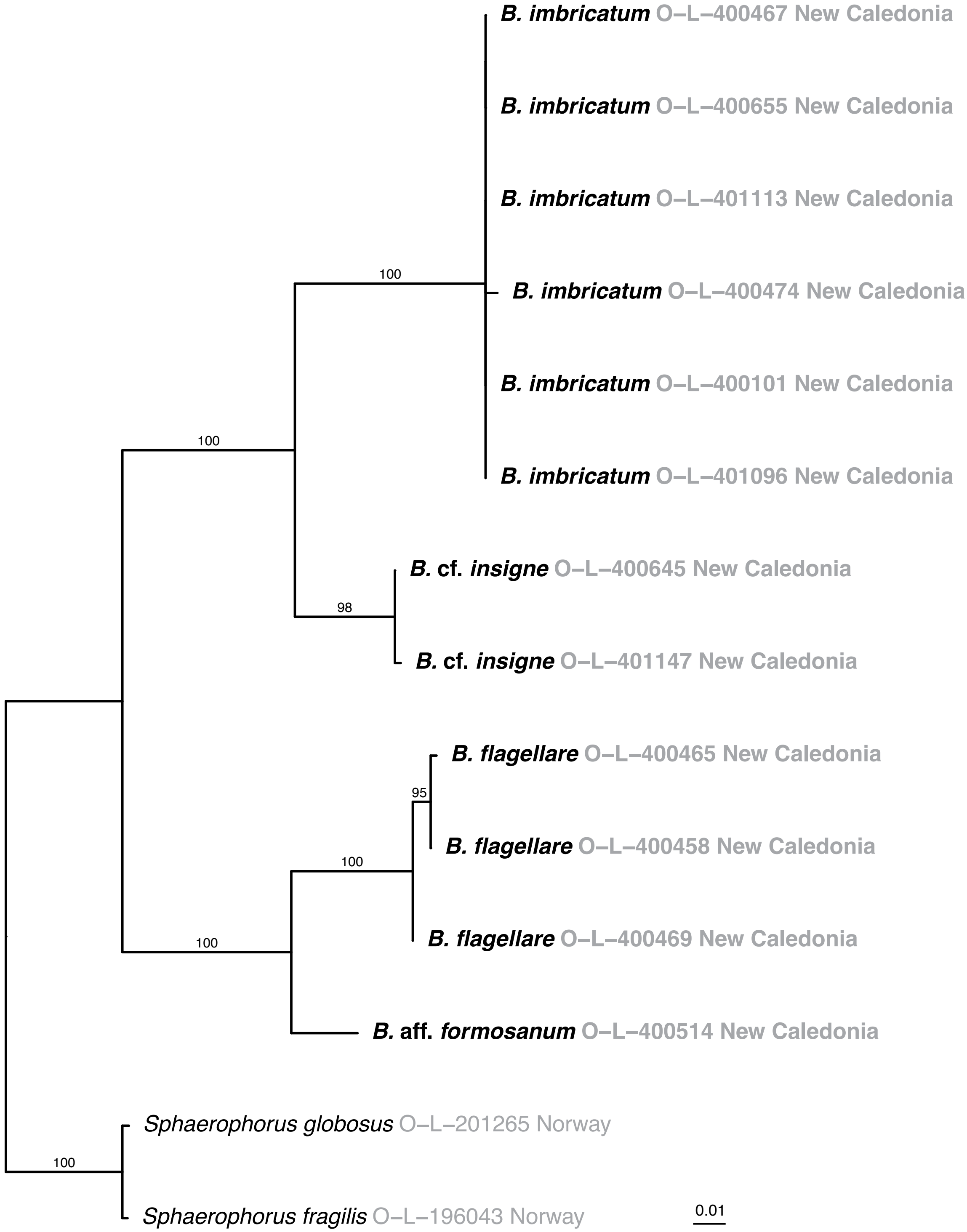

B. imbricatum O–L–400467 New Caledonia
B. imbricatum O–L–400655 New Caledonia
B. imbricatum O–L–401113 New Caledonia
100
B. imbricatum O–L–400474 New Caledonia
B. imbricatum O–L–400101 New Caledonia
100
B. imbricatum O–L–401096 New Caledonia
B. cf. insigne O–L–400645 New Caledonia
98
B. cf. insigne O–L–401147 New Caledonia
B. flagellare O–L–400465 New Caledonia
95
B. flagellare O–L–400458 New Caledonia
100
B. flagellare O–L–400469 New Caledonia
100
B. aff. formosanum O–L–400514 New Caledonia
Sphaerophorus globosus O–L–201265 Norway
100
Sphaerophorus fragilis O–L–196043 Norway
0.01
